# Characterization of cell lines persistently infected with infectious pancreatic necrosis virus

**DOI:** 10.1099/jgv.0.002283

**Published:** 2026-06-18

**Authors:** Lise Chaumont, Mathilde Peruzzi, François Huetz, Claudine Raffy, Jérôme Le Hir, Jules Minke, Pierre Boudinot, Bertrand Collet

**Affiliations:** 1 Université Paris-Saclay, INRAE, UVSQ, VIM, 78350 Jouy-en-Josas, France; 2 Institut Pasteur, Unit of Antibodies in Therapy and Pathology, Inserm UMR1222, Université de Paris, 75015 Paris, France; 3 VIRBAC S.A., Carros, France

**Keywords:** CHSE-EC, interferon response, IPNV, oscillation, persistent infection

## Abstract

Infectious pancreatic necrosis virus (IPNV) is a major pathogen of salmonids that is capable of establishing persistent infections, yet the cellular mechanisms underlying viral persistence remain poorly understood. In this study, we characterized two Chinook salmon embryo-derived cell lines – a wild-type and a Protein Kinase R (PKR)-deficient cell line – which were found to be persistently infected with IPNV. Both cell lines displayed hallmark features of viral persistence, including sustained cell growth without cytopathic effect, continuous production of infectious virus over successive passages and resistance to superinfection by homologous IPNV strains and susceptibility to heterologous viruses. Longitudinal analyses over 40 weekly passages revealed pronounced oscillations in extracellular infectious titres, which partially correlated with fluctuations in intracellular viral RNA levels. Despite these variations in viral replication, persistent infection did not trigger a robust type I interferon response, and basal expression of key interferon-stimulated genes was dampened. Notably, the absence of PKR did not alter viral persistence dynamics, indicating that PKR is not a regulator of this process. Taken together, these findings suggest that persistent IPNV employs immune evasion strategies to suppress host innate immune signalling while maintaining low-level replication.

## Introduction

The infectious pancreatic necrosis virus (IPNV) is a non-enveloped dsRNA virus belonging to the family *Birnaviridae*, in the genus *Aquabirnavirus*. It is the aetiological agent of the infectious pancreatic necrosis [1], initially described as acute catarrhal enteritis in salmonids [2]. IPN is an acute and highly infectious viral disease that causes substantial mortality in salmonid juveniles, particularly in smolts shortly after transfer to seawater [3].

Although IPNV is primarily associated with acute infections, the virus has also been isolated from fish that survive IPN outbreaks and subsequently become asymptomatic carriers. This indicates that IPNV can establish persistent infections, likely by subverting the host defences, enabling the virus to be maintained for extended periods, potentially throughout the host’s lifetime [4,5]. It has been reported that it is able to hide and multiply within head kidney-derived leucocytes from carrier fish without causing lytic infection, suggesting that these cells could be a site of persistent infection [6]. Later, it was shown that both virulent and avirulent strains cause persistent infections [7]. *In vitro*, monolayers of immortalized fish cell lines can become persistently infected by IPNV [7,8]. Several persistently IPNV-infected fish cell lines have been isolated: EPC [9,10], black carp *Myopharyngodon piceus* SB cell line [11], Chinook salmon embryo (CHSE)-214 and STE-137 [12], RTG2 and RTG-P1 [13], as well as fathead minnow EPC [10,14].

Most of these persistently IPNV-infected fish cell lines presented all the canonical characteristics of persistently infected cells [15], including no morphological difference from normal virus-free cells, continuous production of infectious virions over passages, detection of viral antigens in cells, resistance to superinfection with IPNV and susceptibility to heterologous viruses [10,13, 16,20].

The underlying molecular and immunological mechanisms involved in the establishment and maintenance of IPNV persistence both *in vivo* and *in vitro* remain largely unknown. It is likely that persistence results from a tight balance between viral replication and host defence mechanisms. So far, several non-exclusive mechanisms resulting in the establishment of viral persistence have been proposed, including evasion and/or modulation of the host’s immune response, generation of defective interfering particles (DIPs) and modulation of other host pathways [15,19].

During the development and characterization of a *pkr^-/-^* CHSE-derived cell line [21], viral contamination was detected in the supernatants from cells that were theoretically considered non-infected. Notably, no cytopathic effect (CPE) was observed on those cells, suggesting that the cells were either infected with a non-lytic virus and/or harboured a persistent infection. Further real-time quantitative PCR (RT-qPCR) studies confirmed it was an IPNV strain, likely IPNV 31.75 [22]. To date, when and how this persistent infection occurred remains a mystery. This study aimed to characterize these persistently infected cells across successive passages over a long time period (40 weekly passages), assess the stability of the viral persistence over time and examine the impact on the cellular innate immune response.

## Methods

### Cell lines, culture conditions and viruses

The Chinook salmon (*Oncorhynchus tshawytscha*) embryo (CHSE-214) cell line was maintained in Glasgow’s modified Eagle’s medium (GMEM) containing 25 mM HEPES (Biosera) supplemented with 10% FBS (Capricorn Scientific), 2 mM l-glutamine (Eurobio) and penicillin (100 U ml^−1^)–streptomycin (100 µg ml^−1^) (BioValley). The CHSE-EC cell line as well as its derivatives were grown as described previously [23].

IPNV, isolate 31.75, at passage 4 [22], was propagated in CHSE-214 (Mutilplicity of Infection [MOI] 0.001); briefly, the virus was adsorbed onto the cells for 1 h at 14 °C with regular gentle shaking; GMEM supplemented with 2% heat-inactivated FBS was added afterwards. The supernatants were collected at 3–4 days post-infection, 0.2-µm-filtered, diluted 1 : 5 (v/v) in TEN buffer (10 mM Tris, 1 mM EDTA, 150 mM NaCl, pH 7.1) and mixed again 1 : 1 (v/v) in glycerol 100%, aliquoted and stored at −20 °C. IPNV titres were determined by plaque assay on CHSE-214, as described in the ‘Virus titration by plaque assay’ section.

### Development of a *pkr*^-/-^ cell line

A *pkr^-/-^* clone, named EC-PKR-C4^initial^, was obtained following the same procedure as described previously [21]. After propagation of the theoretically clonal cells, it was observed that two populations of cells were present: a majority of GFP-negative cells and a few contaminating GFP-positive cells. The two cell populations were sorted in bulk with a BD FACSAria Fusion Flow Cytometer (Institut Pasteur, Paris, France) using a 100 µm nozzle into two distinct 15 ml tubes. After sorting, cells were centrifuged at 400 ***g*** for 5 min, resuspended and resuspended in L-15 supplemented with 10% FBS, penicillin (100 U ml^−1^)–streptomycin (100 µg ml^−1^), 500 µg ml^–1^ G418 and 30 µg ml^−1^ hygromycin B gold, transferred to 75 cm² flasks and incubated at 20 °C.

The genotype at the sgRNA-targeted site in exon of *pkr* gene (LOC112253229, LG24), of these two cell populations called EC-PKR-C4-GFP(-) and EC-PKR-C4-GFP, had a 1 nt insertion (29_30insT resulting in V11fsX22, KO) and a wild-type (WT) genotype, respectively, as determined previously [21]. The *pkr^-/-^* or WT status of both cell lines was confirmed by western blot using *Salmo salar* IFNA2 supernatant as inducer of *pkr*, as described previously [21].

### Weekly cell passages, supernatant collection and cell sampling

During the characterization process of these two cell lines, it was fortuitously discovered that the two cell lines WT EC-PKR-C4-GFP(+) and *pkr^-/-^* EC-PKR-C4-GFP(-) were persistently infected with IPNV. For convenience purposes, EC-PKR-C4-GFP(+) and EC-PKR-C4-GFP(-) will be referred to as EC^IPNV^ and EC-PKR-C4^IPNV^ thereafter.

**Fig. 1.**
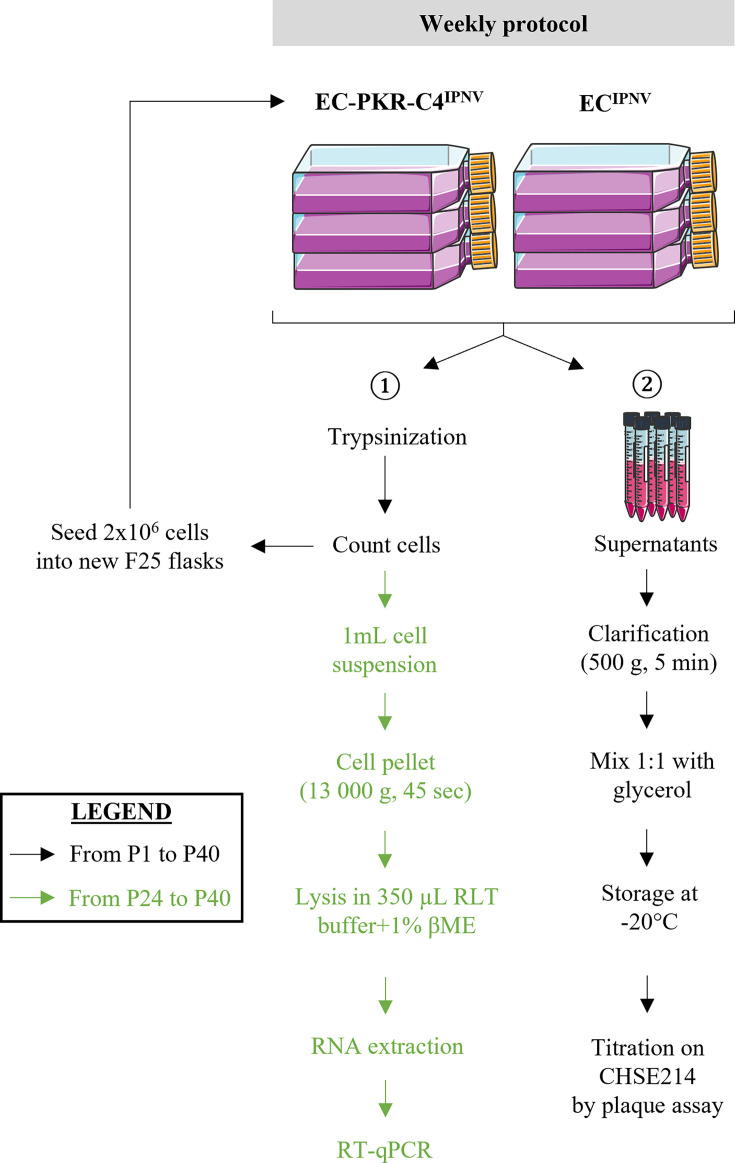
Diagram illustrating the weekly protocol carried out on EC-PKR-C4^IPNV^ and EC^IPNV^. After initial seeding, each flask was treated independently. β-ME, β-mercaptoethanol.

To study the evolution of IPNV persistence over time, EC^IPNV^ and EC-PKR-C4^IPNV^ were maintained in culture for 40 weeks and subcultured weekly, as described in Fig. 1. Initially, EC^IPNV^ and EC-PKR-C4^IPNV^ were seeded into 25 cm² flasks in triplicates to a final density of 2×10^6^ cells/flask in 4 ml L15 + 10% FBS + P/S. For each cell line, the three flasks were then processed independently throughout the rest of the experiment. Each week (i.e. 7 days post-seeding), 1.2 ml of supernatant was harvested from each flask and clarified at 400 ***g*** for 5 min. One millilitre of clarified supernatant was mixed 1 : 1 (v/v) in sterile glycerol (Sigma-Aldrich) and stored at −20 °C until virus titration. In parallel, cells were washed once in Dulbecco's phosphate-buffered saline (Sigma-Aldrich) and trypsinized in 1 ml Alsever’s Trypsin (Gibco)-Versen (Merck) solution (ATV). Once detached, cells were resuspended in 3 ml L15 + 10% FBS + P/S, centrifuged at 400 ***g*** for 5 min. The cell pellet was resuspended in 3 ml L15 + 10% FBS + P/S, and cells were counted using a cell counter (Countess 3, Thermo Fisher) and 2×10^6^ cells were seeded into a new 25 cm² flask. For the last 17 passages (P24–P40), 1 ml of the remaining cell suspension was centrifuged at 13,000 ***g*** for 45 s; the cell pellets were drained, resuspended in 350 µl RLT buffer (QIAGEN) supplemented with 1% β-mercaptoethanol and stored at −80 °C until use for RNA extraction.

### Virus titration by plaque assay

IPNV titres were determined by plaque assay on CHSE-214 cells. CHSE-214 cells were seeded into 12-well plates at a final density of 7

×10^5^ cells/well in GMEM + 2% FBS + P/S. The next day, the harvested supernatants (previously stored at −20 °C) were 10-fold serially diluted in L-15 + 2% decomplemented FBS + P/S, and 100 µl of each dilution was applied onto the cells in technical duplicates. After a 1-h-long adsorption phase, a carboxymethylcellulose overlay [0.75% in MEM (Eurobio) supplemented with 25 mM HEPES, 2 mM l-glutamine, 350 mg l^−1^ NaHCO_3_, 2.5% FBS and P/S] was added onto the cells. At 7 days post-infection, the cells were fixed with 3.7% formaldehyde (Sigma-Aldrich) for 1 h at room temperature, stained with 0.5% crystal violet (Sigma-Aldrich) and plaque-forming units (p.f.u.) were counted.

### Real-time quantitative PCR

Total RNA was extracted from cells in RLT buffer using QiaShredder and RNeasy mini kits (QIAGEN) in accordance with the manufacturer’s instructions. In addition to the persistently infected cell samples, positive and negative controls were also included. For this purpose, EC (WT) cells were seeded into six-well plates to a final density of 1.5×10^6^ cells/well in L-15 + 2% FBS + P/S. The next day, cells in triplicates were infected with IPNV 31.75 (MOI 1) or left untreated and incubated at 14 °C for 8 hpi or 24 hpi; in parallel, cells were stimulated with recombinant *S. salar* IFNA2 supernatant (produced as described previously) [21] diluted to 1 : 10 in L-15 + 2% FBS + P/S for 72 h or left untreated. Cells were collected at indicated timepoints, and total RNA was extracted as described above. For all samples, quality control of the extracted RNA was determined using a NanoDrop spectrophotometer.

The cDNA was generated from 2.25 µg of total RNA using the iScript™ Advanced cDNA Synthesis Kit for RT-qPCR (Bio-Rad), and the synthesis was performed in a thermal cycler (Eppendorf) as recommended by the manufacturer. cDNA was diluted to 1 : 4.5 in DNase- and RNase-free water and stored at −20 °C until use. ‘No RT’ control reactions were made for a few representative samples by omitting the reverse transcriptase. The cDNA was mixed with TB Green qPCR Premix Ex Taq (Tli RNaseH Plus, Takara) along with forward and reverse primers (Table 1) at a final concentration of 210 nM each in Twin.tec^®^ real-time PCR plates (Eppendorf). As all samples did not fit on a single 96-well qPCR plate, an internal calibrator consisting of a pool of all samples was added onto each plate. Amplification was performed using a CFX Connect cycler (Bio-Rad) using the following cycling programme: initial denaturation at 95 °C for 30 s, followed by 40 cycles of 10 s at 95 °C and 30 s at 60 °C. For each biological replicate, mean Cq values of target genes were calculated based on technical duplicate reactions and then normalized using the geometric mean of Cq values of three housekeeping genes (*otelf1α, otrps29, otgapdh3*). The relative expression of each target gene (*ipnv*, *otirf1*, *otirf3*, *otmx123, otpkr*) was expressed as 2^−∆Cq^.

**Table 1.** qPCR primers used in this study

Target gene name	Sequence 5’−3**’**	Target accession no./ size (bp)/efficiency or reference		
Elongation factor 1-α, oocyte form	CACTGCTCAAGTAATCATCCTG	[21]		
	CACAGCAAAACGACCAAGAG			
Glyceraldehyde-3-phosphate dehydrogenase	CCAGTGTATGAAGCCCCATGAG			
	CTTGTCCTCGTTGACTCCCATG			
40S ribosomal protein S29	GGGTCATCAGCAGCTCTATTGG			
	CCAGCTTAACAAAGCCGATGTCG			
Interferon-induced GTP-binding protein Mx1 Mx2 Mx3	CAACTTGGTGGTTGTGCCATG			
	GGCTTGGTCAGGATGCCTAAT			
Protein kinase R, full-length isoform	CTGAGTAAAGGGAAAGCTAAGCGG			
	GCCTGAATCTGAAGTGGTGTCG			
IPNV segment A pVP2-VP4-VP3	CCTTGACAATGACGTCCCAGTG	[42]		
	GACTGGGTCATCTTGGCTGAG			
Interferon regulatory factor 3	CAAGGCGTGGGCTGAGG	[43]		
	CTGGGTGCTGAGATCCTCCTG			
Interferon regulatory factor 1-like	CCACCCCACAGACTATGAAGAC	XM_024432221	191	This study
	GCTCTATTTCCGCCCCTGAG			

For each set of primers, the efficiency was calculated by linear regression obtained by using 10-fold serial dilutions of plasmid containing the target sequence (*otmx123*, *otpkr*) or of a pool of cDNA (housekeeping genes, *ipnv*, *otirf3*, *otirf1*) and the qPCR products were validated by gel migration and sequencing.

### Viral permissivity test

WT EC, *pkr^-/-^* EC-PKR-C19, WT EC^IPNV^, *pkr^-/-^* EC-PKR-C4^IPNV^ cell lines were seeded in 96-well plates at a density of 7×10^4^ cells/well in L-15 + 2%FPS + P/S and incubated overnight at 20 °C. The next day, the cells were infected with 10-fold serial dilutions of either IPNV (isolate 31.75, stock at 2.2×10^8^ p.f.u. ml^–1^) [22] or infectious hematopoietic necrosis virus (IHNV, isolate 25.70, stock at 1.1×10^6^ p.f.u. ml^–1^) [24]. The first wells were infected at different initial MOI depending on the virus (IPNV: MOI 0.005; IHNV: MOI 0.3). At 7 days post-infection, cells were fixed with 3.7% formaldehyde for 1 h and stained with 0.5% crystal violet.

### Statistical analysis

The data presented are means±sd. Statistical tests used are indicated in the legend of each figure. All statistical analyses and the sinusoidal model fitting were performed using GraphPad Prism software version 8.0.1.

## Results

### Development of an initial *pkr*^-/-^ cell line and identification of a persistent IPNV infection

**Fig. 2.**
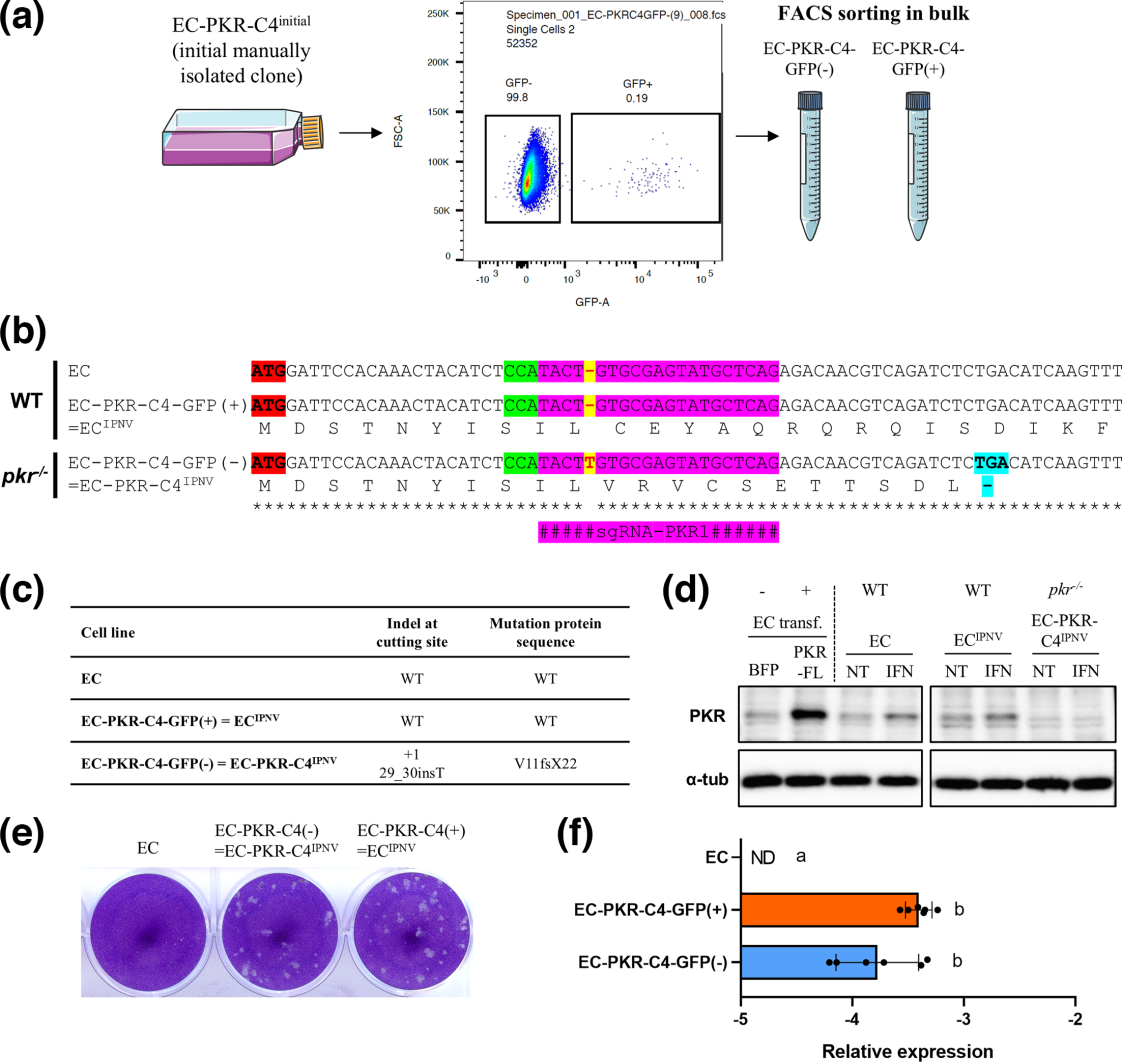
Validation of the PKR expression status in EC-PKR-C4-GFP(-) and EC-PKR-C4-GFP(+) and identification of a persistent IPNV infection in both cell lines. (**a**) Diagram illustrating the FACS-based isolation process of EC-PKR-C4-GFP(-) and EC-PKR-C4-GFP(+) from an initial manually isolated clone called EC-PKR-C4^initial^ presenting a discrete population of GFP-positive cells. (**b**) Genotype of EC cells (WT), latently IPNV-infected EC-PKR-C4-GFP(+) (WT, referred to as EC^IPNV^) and EC-PKR-C4-GFP(-) (1 nt insertion, 29_30insT resulting in V11fsX22, pkr^-/-^, referred to as EC-PKR-C4^IPNV^) obtained from sequencing of purified PCR products amplified from genomic DNA from each cell line. The location of the sgRNA is highlighted in pink, the protospacer adjacent motif is in green, the start codon is in red, the 1 nt insertion in EC-PKR-C4-GFP(-) is in yellow and the premature stop codon is in blue. The corresponding chromatograms are available in Fig. S1 (available in the online Supplementary Material). (**c**) Table summarizing the molecular characteristics of EC, EC^IPNV^ and EC-PKR-C4^IPNV^ cell lines. (**d**) EC, EC^IPNV^ and EC-PKR-C4^IPNV^ cells were stimulated with *S. salar* IFNA2 supernatant for 72 h; positive and negative controls are EC cells transfected with pcDNA3.1-Hyg-BFP or pcDNA3.1-Zeo-Hyg-P2A-PKR-FL, respectively. Cell lysates were separated by SDS-PAGE and immunoblotted with antibodies against *S. salar* PKR and α-tubulin (α-tub). Full-length blots are available in Fig. S2. Blots are representative of two independent experiments. (**e**) CHSE-214 were incubated with supernatants from EC, EC-PKR-C4(+) and EC-PKR-C4(-) under a carboxymethylcellulose overlay at 14 °C for 7 days, fixed with formaldehyde and stained with crystal violet. (**f**) Relative expression levels of IPNV transcripts in non-infected EC cells, EC-PKR-C4(+) and EC-PKR-C4(-). Graph shows means±sd from two independent experiments (*n*=6); letters indicate significant differences between cell lines, ordinary one-way ANOVA with Tukey’s post hoc multiple-comparison test.

To characterize the function of Chinook salmon PKR, the unique *pkr* gene (LOC112253229, LG24) was disrupted in EC cells using CRISPR/Cas9 genome editing technology. Four manually isolated clones were initially obtained, but only one of them, called EC-PKR-C4^initial^, presented a mutated genotype at the targeted cut site (data not shown). However, observations under the fluorescence microscope revealed the presence of a few contaminating GFP-positive fluorescent cells. The GFP-positive and -negative populations were FACS-sorted in bulks; the resulting cell lines were called EC-PKR-C4-GFP(+) and EC-PKR-C4-GFP(-), respectively (Fig. 2a). Genotyping results showed that EC-PKR-C4-GFP(+) cells had a WT genotype, while EC-PKR-C4-GFP(-) presented a 1 nt insertion at the targeted cut site, leading to a frameshift resulting in the introduction of a premature codon at position 22 (29_30insT resulting in V11fsX22, Fig. 2b, c). The PKR expression status in both cell lines was assessed at the protein level by western blot using IFNA2 supernatant as an inducer of *pkr* expression. Our results showed that PKR was induced in WT EC and WT EC-PKR-C4-GFP(+) cells following IFNA2 treatment. In contrast, no PKR signal was detected in *pkr^-/-^* EC-PKR-C4-GFP(-) cells, thereby confirming that the expression of PKR was effectively abolished in these cells (Fig. 2d). EC-PKR-C4-GFP(+) was kept as an additional positive control for further experiments.

During the characterization process of *pkr^-/-^* EC-PKR-C4-GFP(-) [along with EC-PKR-C4-GFP(+)], it was observed that supernatants from healthy cells showing no visible signs of CPE were able to induce CPE on CHSE-214 cells (Fig. 2e). Further RT-qPCR experiments revealed that both cell lines expressed IPNV transcripts (Fig. 2f). In addition, preliminary titration results indicated that infectious virion particles were present in the supernatants of both cell lines (10^3^–10^4^ p.f.u. ml^–1^) (data not shown). These findings showed that both cell lines were persistently infected with IPNV. WT EC-PKR-C4-GFP(+) and *pkr^-/-^* EC-PKR-C4-GFP(-) were renamed EC^IPNV^ and EC-PKR-C4^IPNV^, and this denomination will be used thereafter. In addition, even EC-PKR-C4^initial^ was found to be persistently infected (data not shown), suggesting that the event leading to the establishment of IPNV persistence occurred prior to FACS sorting.

### Characterization of a persistent IPNV infection in *pkr*^-/-^ EC-PKR-C4-GFP(-) and WT EC-PKR-C4-GFP(+)

We decided to characterize the IPNV persistence in both cell lines over the course of passages. The aim was to study whether IPNV persistence was changing over time and investigate whether PKR was modulating this phenomenon. To this end, EC^IPNV^ and EC-PKR-C4^IPNV^ were maintained in culture for 40 weeks. At each weekly passage (P1–P40), supernatants from three independent flasks for each cell line were collected and IPNV titres were determined by plaque assay. For the last 17 passages, cell samples from the same flasks were also collected for RT-qPCR analysis (Fig. 2)

### The extracellular titres of persistent IPNV oscillate over the course of passages in both cell lines

**Fig. 3.**
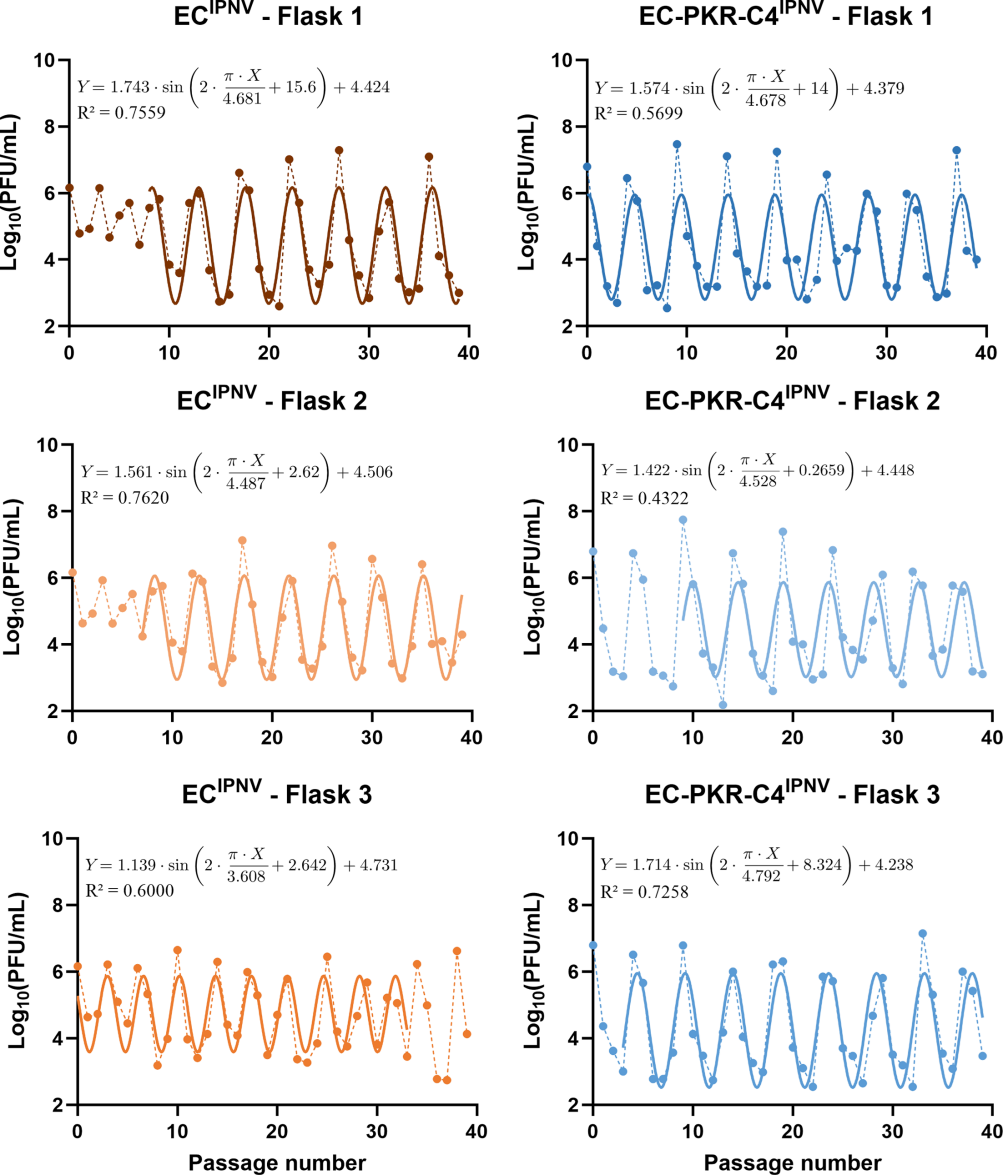
Extracellular viral titres from supernatants of EC^IPNV^ and EC-PKR-C4^IPNV^ cells persistently infected with IPNV. For (**a**) EC^IPNV^ and (**b**) EC-PKR-C4^IPNV^, supernatants from three independent flasks were collected prior to weekly subcultures and titres were determined by standard plaque assay. Each coloured line represents an individual flask. A sinusoidal model was fitted to each data series. The model used was a sine wave with non-zero baseline with the following equation: *Y*(*X*) = *A* sin((2π*X*/λ) + φ) + *B*, where *A* is the amplitude, λ is the wavelength, φ is the phase shift (radians) and *B* is the baseline. The solid line represents the fitted model, and the dotted line represents the measured extracellular titres. p.f.u., plaque-forming unit.

Titration of supernatants from EC^IPNV^ and EC-PKR-C4^IPNV^ collected over 40 passages shows regular oscillations in extracellular titres in each individual flask for both cell lines (Fig. 3). In both cases, extracellular titres range from 10^2^ to 10^8^ p.f.u. ml^–1^. By comparison, extracellular titres obtained with an acute and lytic IPNV infection typically range from 10^8^ to 10^9^ p.f.u. ml^–1^ in CHSE-214 [25] and EC cells. A sine model was fitted to the experimental data and the wavelength (period) was estimated. The extracellular titres oscillate with a mean period of T=4.26±0.57 passages for EC^IPNV^ and T=4.67±0.13 passages for EC-PKR-C4^IPNV^ (NS, unpaired t-test).

**Fig. 4.**
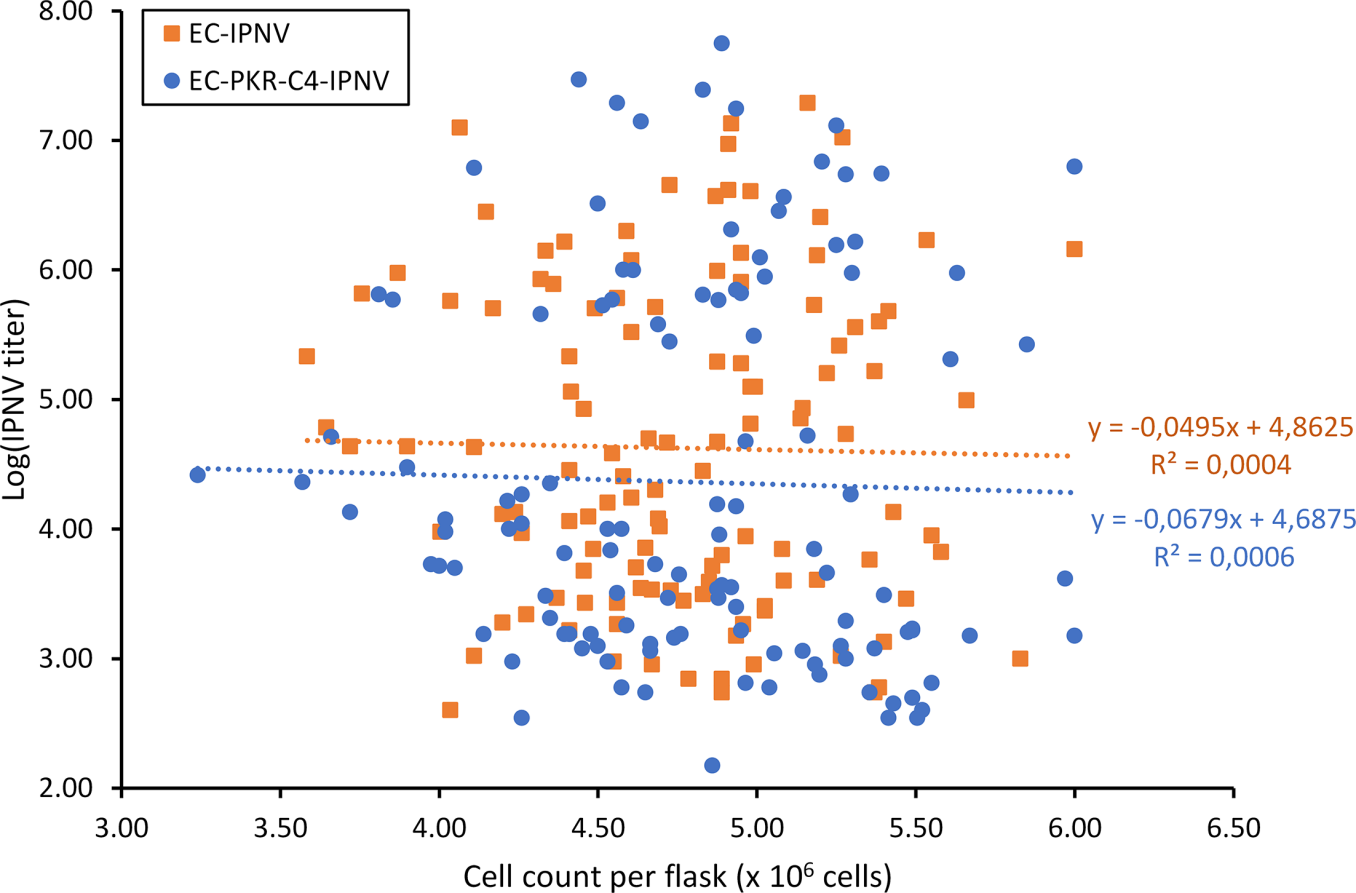
Correlation between the number of cells per flask and the extracellular viral titres in the supernatants at each weekly sampling timepoint in EC^IPNV^ and EC-PKR-C4^IPNV^.

To confirm that these fluctuations are not due to a storage issue, a few samples were randomly selected and titrated a second time, yielding similar titres as the ones previously obtained (data not shown). In addition, extracellular titres do not correlate with the number of cells in each flask at each sampling time, indicating that extracellular titres are not caused by fluctuations in cell growth over time (Fig. 4).

### Oscillations in extracellular titres are explained by intracellular replication fluctuations

**Fig. 5.**
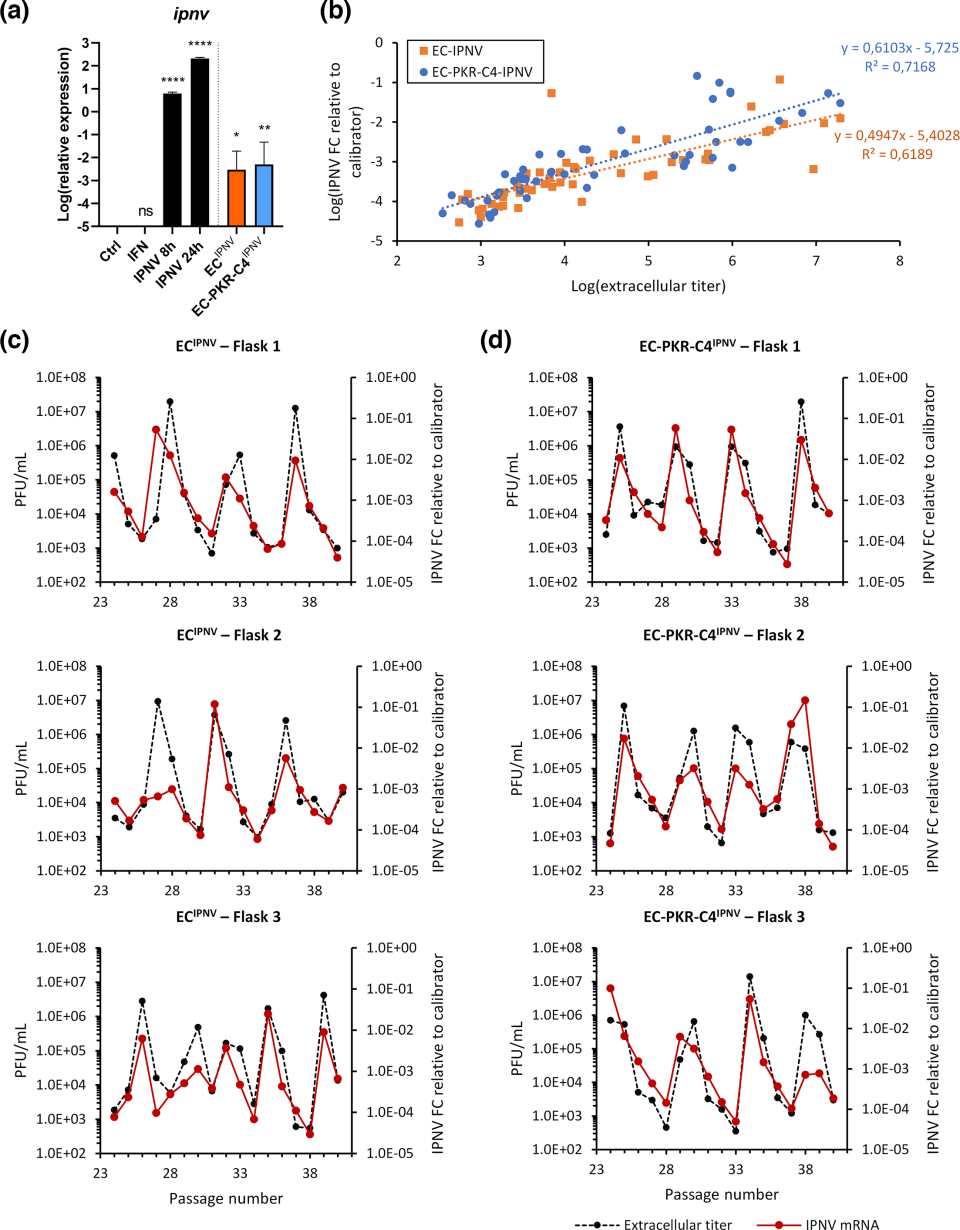
Intracellular IPNV replication correlates with extracellular titres. (a) Black bars show means ± sd of relative expression levels of IPNV transcripts in EC cells infected with IPNV 31.75 (MOI 1) for 8 and 24 h post-infection, stimulated with recombinant type I IFN (72 h) or left untreated (*n*=3 for each condition); ns, non-significant, ****P<0.0001, ordinary one-way ANOVA with Tukey’s post hoc multiple-comparison test. Coloured bars show means ± sd of relative expression levels of *ipnv* mRNA in persistently IPNV-infected WT EC^IPNV^ and *pkr*^-/-^ EC-PKR-C4^IPNV^ (for each cell line, *n*=3×17, corresponding to all the samples collected from P24 to P40). *P<0.05, **P<0.01, Kruskal–Wallis test with Dunn’s post hoc multiple comparison test. (b) Dotplot showing log-transformed *ipnv* mRNA levels (represented as fold change relative to calibrator) as a function of log-transformed extracellular titres in persistently IPNV-infected EC^IPNV^ and EC-PKR-C4^IPNV^ cells. Distinct linear regressions were performed on EC^IPNV^ and EC-PKR-C4^IPNV^ datasets. (c, d) Graphs showing extracellular titres and *ipnv* mRNA levels over the course of passages in each individual flasks of EC^IPNV^ (c) and EC-PKR-C4^IPNV^ (d).

To identify whether the fluctuations in extracellular titres were due to variations in virus replication, EC^IPNV^ and EC-PKR-C4^IPNV^ cell samples were collected from P24 to P40 during the passage process and the extracted RNA was analysed by RT-qPCR using primers targeting *pVP2-VP4-VP3* gene. EC cells infected with IPNV 31.75 or left uninfected were used as positive and negative controls, respectively. Our results revealed that both WT EC^IPNV^ and *pkr^-/-^* EC-PKR-C4^IPNV^ continuously expressed IPNV mRNA over passages, but it was at a much lower level than acutely IPNV-infected EC cells (3-log difference at 8 hpi and almost 5-log difference in relative expression at 24 hpi compared to IPNV-infected EC cells, Fig. 5a).

Interestingly, IPNV mRNA expression is positively correlated with the corresponding extracellular titres in both cell lines with robust coefficients of determination (*R*²=0.62 for EC^IPNV^ and *R*²=0.71 for EC-PKR-C4^IPNV^) (Fig. 5b). Consistently, expression fluctuations in individual flasks for both cell lines over passages show regular oscillations with peaks in viral mRNA expression occurring at the same time as peaks in extracellular titres (Fig. 5c, d). However, it seems that the ‘peak intensity’ in viral transcripts does not necessarily systematically match extracellular titre levels, suggesting that other factors involved in modulation of replication, viral mRNA degradation and/or virion release might be at play.

These findings suggest that oscillations in extracellular titres in both cell lines can be (at least partially) explained by fluctuations in intracellular replication.

### The innate immune response is dampened in persistently IPNV-infected cell lines

**Fig. 6.**
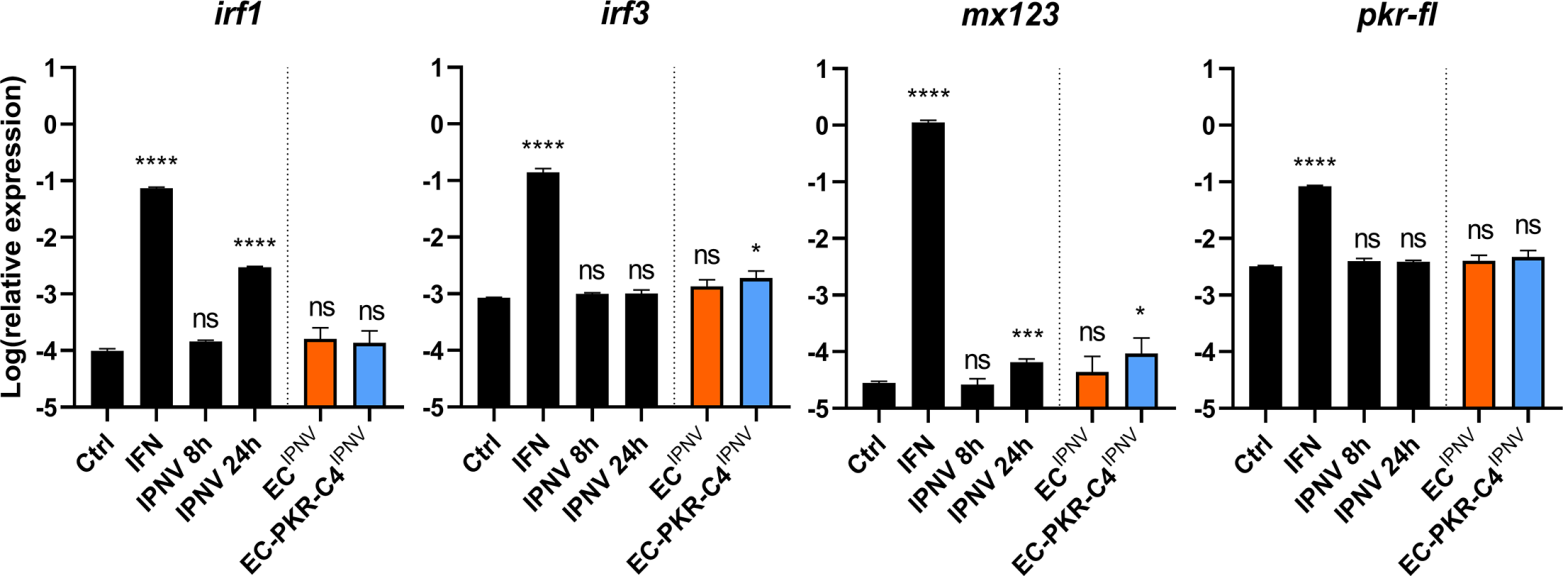
Persistent and acute IPNV infections dampen the innate immune response in infected cells. Black bars show means±sd of relative expression levels of target genes (*irf1*, *irf3*, *mx123*, *pkr-fl*) in EC cells infected with IPNV 31.75 (MOI 1) for 8 and 24 h post-infection, stimulated with recombinant type I IFN (72 h) or left untreated (*n*=3 for each condition); ns, non-significant, ***P<0.001, ****P<0.0001, ordinary one-way ANOVA with Tukey’s post hoc multiple-comparison test. Coloured bars show means±sd of relative expression levels in persistently IPNV-infected WT EC^IPNV^ and *pkr*^-/-^ EC-PKR-C4^IPNV^ (for each cell line, *n*=3×17, corresponding to all the samples collected from P24 to P40). *P<0.05, Kruskal–Wallis test with Dunn’s post hoc multiple-comparison test.

In order to investigate whether IPNV persistent infection modulates the expression of innate immune genes, the expression profiles of selected *ISGs* (*irf1*, *irf3*, *mx123*, *pkr*) were examined in EC^IPNV^ and EC-PKR-C4^IPNV^ over the course of passages. EC cells simulated with recombinant type I IFNA2 supernatant or left untreated were used as positive and negative controls, respectively. EC cells acutely infected with IPNV 31.75 for 8 or 24 hpi were also included for comparison purposes. For all four genes, a strong and significant induction in mRNA expression was observed following type I IFN treatment compared to non-stimulated cells, thereby validating the assay and the *ISG* status of the selected genes. In contrast, while *irf1* expression was induced at 24 h post-infection with IPNV in EC cells, *mx1* only displayed a weak induction at the same timepoint and *irf3* and *pkr* transcripts were not induced upon IPNV infection at any of the timepoints examined (Fig. 6, black bars). These results suggest that acute infection with IPNV suppresses the early activation of specific immune genes. Interestingly, similar results were previously reported in rainbow trout RTG-P1 cells, which showed inhibition in *mx* expression following IPNV infection [26].

In EC^IPNV^ and EC-PKR-C4^IPNV^, the mean expression of *irf1* and *pkr* was not modulated compared to non-infected cells. Interestingly, *irf3* and *mx123* were overall weakly but significantly more expressed in EC-PKR-C4^IPNV^ compared to EC^IPNV^ (Fig. 6, coloured bars). Taken together, these results suggest that, in a similar fashion to acute IPNV infection, the host innate immune response is overall dampened during persistent infection.

**Fig. 7.**
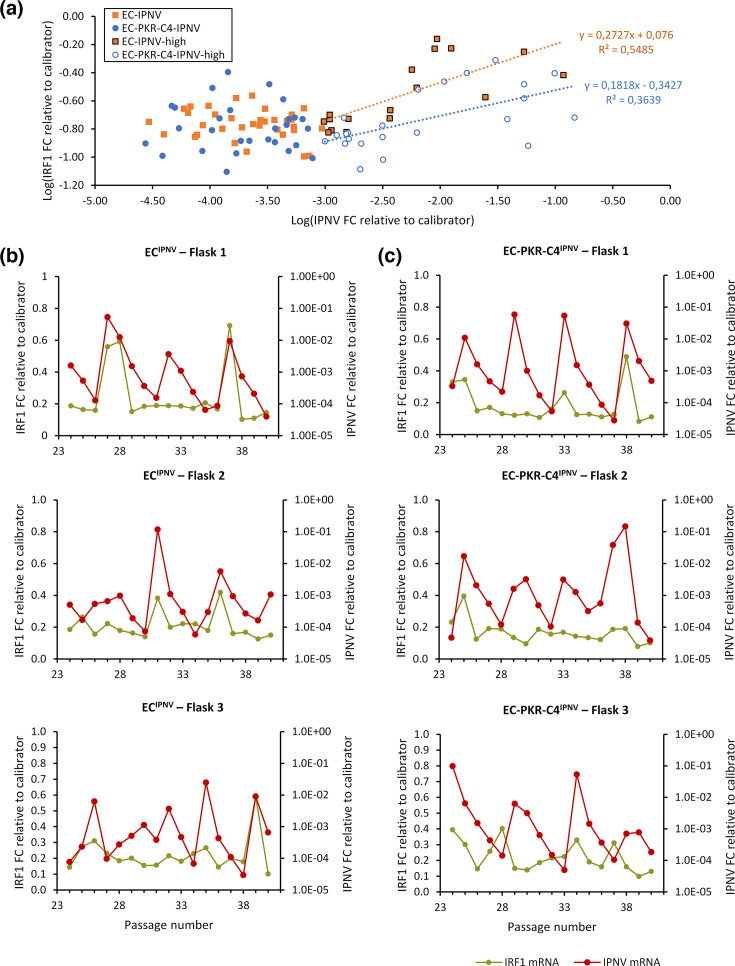
IRF1 is induced in both in both acutely and persistently infected cells. (**a**) Dotplot showing log-transformed *ipnv* mRNA levels (represented as fold change relative to calibrator) as a function of log-transformed *irf1* mRNA levels in persistently IPNV-infected EC^IPNV^ and EC-PKR-C4^IPNV^ cells. Linear regressions were performed on EC^IPNV^ and EC-PKR-C4^IPNV^ subsets expressing high levels of *ipnv* mRNA (threshold log(IPNV FC relative to calibrator) > −3). (**b, c**) Graphs showing *ipnv* and *irf1* mRNA levels over the course of passages in each individual flasks of EC^IPNV^ (**b**) and EC-PKR-C4^IPNV^ (**c**).

These results do not show, however, the variations in expression that may occur over time. For both cell lines, the expression profiles of each Interferon-Stimulated Gens (ISG) were examined in individual flasks over passages in comparison with IPNV mRNA expression results. For *irf3*, *mx123* and *pkr*, variations in expression over time do not correlate with the corresponding viral mRNA and no other expression pattern could be identified (Figs S3–S5). In contrast, *irf1* transcript levels are somewhat correlated with viral mRNA levels (Fig. 7). In particular, expression variations in individual flasks for both cell lines show a few peaks in *irf1* expression occurring at the same time as peaks in viral transcripts (Fig. 7b, c). Consistently, sample subsets expressing high levels of viral mRNA display a positive correlation between *irf1* expression and viral gene expression. These results suggest that *irf1* expression may be triggered when viral transcript levels are over a specific threshold, hence a correlation only visible in samples expressing high levels of viral mRNA (Fig. 7a). These findings are also reminiscent of the results obtained in the case of acute infection, which induced *irf1* expression but not the expression of other innate immune genes (Fig. 6).

### Persistently IPNV-infected cells are refractory to acute IPNV infection but are still permissive to other viruses

**Fig. 8.**
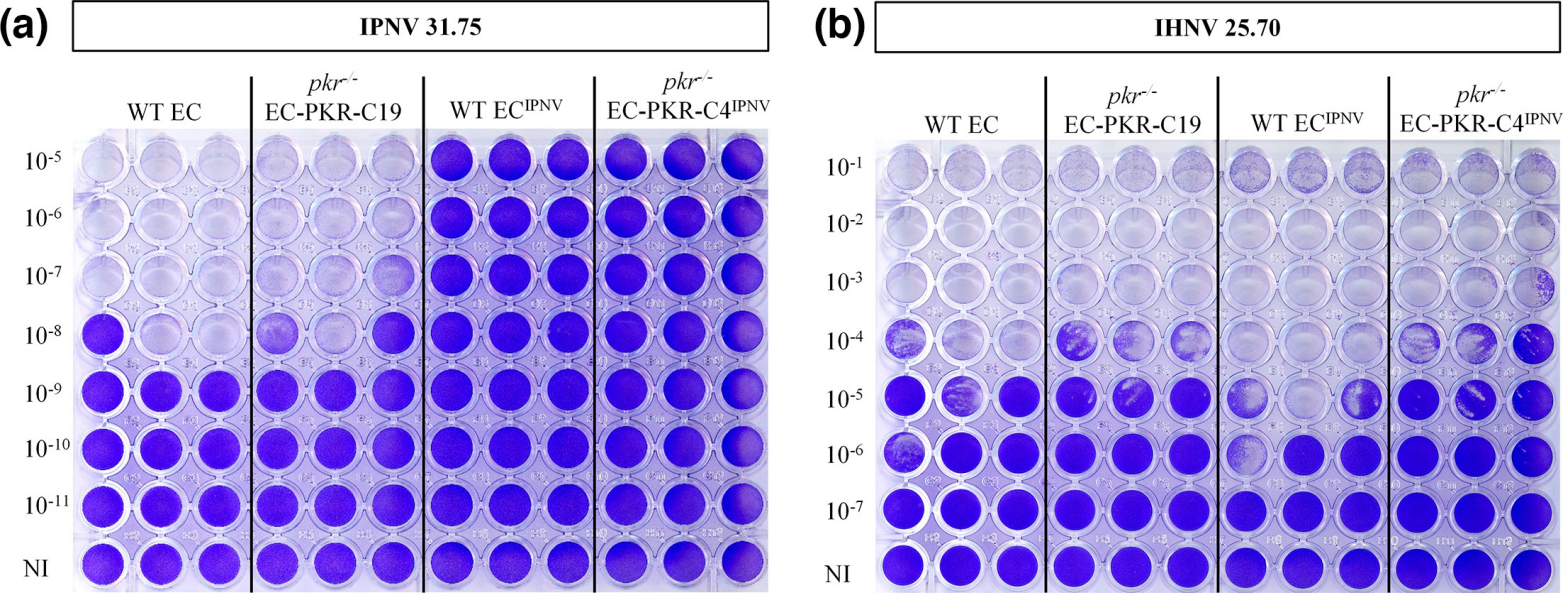
Comparison of permissivity to IPNV 31.75 and IHNV 25.70 of IPNV-free cell lines and persistently IPNV-infected cells. WT EC, *pkr^-/-^* EC-PKR-C19, WT EC^IPNV^, *pkr^-/-^* EC-PKR-C4^IPNV^ were infected with 10-fold serial dilutions of (**a**) IPNV 31.75 (starting MOI=0.005) or (**b**) IHNV 25.70 (starting MOI=0.3) and incubated for 7 dpi at 14 and 22 °C, respectively.

We investigated the permissivity of EC^IPNV^ and EC-PKR-C4^IPNV^ to viral infections, including IPNV strains as well as heterologous viruses. Persistently infected EC^IPNV^ and EC-PKR-C4^IPNV^ cell lines were refractory to acute infection with IPNV 31.75 and displayed no signs of CPE contrary to their IPNV-free counterparts (Fig. 8a). Comparable results were obtained with infection at higher MOIs (up to MOI=50) as well as with other IPNV strains, including IPNV TA and IPNV PT (data not shown). In contrast, EC^IPNV^ and EC-PKR-C4^IPNV^ showed no difference in permissivity following infection with IHNV 25.70 compared to non-persistently infected cells (Fig. 8a). Similar observations were made following infection with other heterologous viruses, including Viral Haemorrhagic Septicaemia Virus, VHSV and Epizootic Haematopoietic Necrosis Virus, EHNV (data not shown).

Taken together, these results suggest that persistently IPNV-infected cells are refractory to acute IPNV infection, regardless of the strain/isolate. However, the IPNV persistence does not seem to alter their permissivity to viral infection with heterologous viruses.

## Discussion

In this study, we characterized two Chinook salmon cell lines deriving from the parental EC cell line, a WT cell line and a *pkr^-/-^* cell line, which were found to be persistently infected with IPNV, rendering them refractory to acute IPNV infection. We observed that the extracellular titres of persistent IPNV oscillate with a remarkable regularity over the course of passages in both cell lines and that they correlate – at least partially – with variations in intracellular viral replication. Our results further indicate that the basal expression of key ISG transcripts is dampened during persistent infection, in a similar fashion to what occurs during acute IPNV infection, and that PKR is not primarily involved in the regulation of this persistence phenomenon. Taken together, these results suggest that persistent IPNV has evolved strategies to evade the host innate immune response by inhibiting the expression of innate immune genes, while maintaining a relatively low replication level.

Our persistently infected cell lines present most of the characteristics typically described for viral persistence in mammalian cells, including continued growth of infected cells that are morphologically indistinguishable from virus-free cells, continued production of infectious virions over passages; resistance to superinfection by homologous virus strains and susceptibility to heterologous viruses [15]. One last criterion mentioned by Rima and Martin [15] is the presence of viral antigen in a majority of cells, which was not examined in the present study [15]. These results are fully consistent with other studies on fish cell lines persistently infected with IPNV [10,12, 13, 16, 18,20].

Contrary to our study, no specific patterns in extracellular titres were identified by [16] in persistently IPNV infected CHSE-214 and STE-137 cells, although considerable variations were measured over weekly passages [12,16]. In contrast, [13], noticed that in persistently IPNV-infected RTG-2 cells, extracellular titres fluctuated during 20 passages with a periodicity of 6 passages, following the same pattern as cells expressing viral antigens. Our study was performed over a longer period of time and confirmed that this periodic pattern is, indeed, present in IPNV-infected EC cells. Importantly, similar oscillations in titres from persistently infected cells have been reported in both *in vitro* and *in vivo* mammalian models during persistent infections with Vesicular Stomatitis Virus, VSV or rabies [27,29].

This curious phenomenon prompted us to investigate the underlying mechanisms. For this purpose, we studied what was happening intracellularly in terms of viral replication and host innate immune response. Our results show that, overall, intracellular viral RNA levels follow the same pattern as extracellular infectious titres. However, it appeared that the innate immune response was not triggered by the persistent infection, irrespective of the intracellular IPNV replication levels. These results are in line with early *in vitro* studies, which showed that persistently IPNV-infected CHSE-214 and STE-137 cell lines displayed no IFN-like activity [16,18]. Consistently, no differentially expressed ISGs were found by suppression subtractive hybridization in persistently IPNV-infected CHSE-214 cells compared with non-infected cells [19]. Similar observations were also made *in vivo* in IPNV-carrier Atlantic salmon, which displayed low viral RNA levels and no induction of *mx* in head kidney [30]. These results may be linked to the ability of IPNV to evade the innate immune response even during an acute infection: for instance, IPNV infection was reported to suppress poly(I:C)-induced activation of *mx* promoter in the RTG-P1 reporter cell line [26]. Similarly, IPNV was found to limit *mx* expression in CHSE-214 cells pre-infected with IPNV and stimulated with recombinant IFNa1 [31]. Mechanistically, it seems that IPNV-mediated inhibition of the type I IFN signalling pathway involved VP4 and VP5 but the exact underlying mechanisms are currently unknown [31,32]. In any case, it is possible that persistent IPNV has implemented similar mechanisms of action to block the induction of ISGs and/or IFN genes, enabling it to replicate without killing the host cells.

In addition to directly inhibiting the IFN signalling pathway, other authors have proposed that the *in vivo* persistence status may also be favoured by the virus reducing its replication levels so as not to trigger host defences [30]. Our results on *irf1* expression levels, which seem to be slightly induced in persistently infected cells presenting higher levels of viral RNA (although to a much lower level compared to acute infection), support this hypothesis.

Altogether, these results suggest that persistent IPNV has evolved strategies to evade the host innate immune response and even inhibits the expression of genes that are usually induced following acute infection, such as *irf1*. It appears, however, that variations in viral transcripts may cause inhibition to ‘leak’ when viral transcripts are above a specific threshold, resulting in a few *irf1* expression peaks. In addition, the fact that some viral mRNA expression peaks are not associated with higher *irf1* expression may be due to other viral evasion mechanisms.

In mammalian models, oscillations in extracellular viral titres in cells persistently infected with various RNA viruses were linked to the production of viral DIPs [28,29, 33].

DIPs are viral particles containing ‘normal’ structural proteins but a defective viral genome due to mutations, deletions or gene rearrangements [34]. Most of them are able to enter permissive cells but are not replicative per se due to their truncated genome. However, they can replicate in case of co-infection with a ‘helper’ standard infectious virus to complement the lost functions and give rise to similar progeny DIPs [3,35]. By doing so, they may also interfere with the replication of non-defective WT viruses by competing for viral factors: due to their shortened length, they can replicate more efficiently than WT virus, resulting in their accumulation in co-infected cells [33,35]. In cells persistently infected with VSV or rabies, it was observed that an increase in DIP production was occurring right after the production peak of infectious standard virus [28,29]. A similar periodic pattern was observed *in vivo* in mice co-infected with standard VSV and DIPs with a periodicity of ~5 days [36]. It was proposed that this cyclic production dynamics was similar to a predator–prey relationship [27], suggesting that specific subpopulations may determine the composition of the virus pool in the supernatants [33]. This cycling pattern was later mathematically modelled using input–output models incorporating interactions between standard virions, DIPs and cells to predict yields of infectious virus and DIPs [37,38].

In our study, we did not examine the production of DIPs in persistently IPNV-infected CHSE-EC cells. Nonetheless, a few early studies provide evidence of the presence of DIPs in cells infected with both lytic IPNV and persistent IPNV [16,39]. More specifically, electron microscopy studies revealed the presence of incomplete virions different from the electron-dense ones observed during acute infection [16]. Analysis of persistent virions from infected STE-137 cells by centrifugation in caesium chloride gradients confirmed that two types of virions were produced: high-density infectious virions and low-density defective virions; the latter were able to delay CPE following infection with stock IPNV, suggesting that defective virions were DIPs [16]. In line with these results, CPE-suppressing activity against IPNV but not heterologous viruses (e.g. IHNV, VHSV) was also reported in the supernatants from persistently IPNV-infected RTG-2, suggesting the presence of DIPs [13]. However, whether DIPs oscillate asynchronously with infectious IPNV particles is still unclear. For other viruses, persistently infected cell lines that exhibit oscillations in viral p.f.u. titre have been explained by interactions between WT virions and DIPs [40], and mathematically modelled [41]. The latter model, under particular sets of parameters, predicts that the virus can persist indefinitely while maintaining sustained titre oscillations. Our data strongly support the idea that our persistently infected cell lines conform to such a model, in which periodic, interdependent fluctuations in defective interfering and WT particles drive and stabilize the system’s oscillatory behaviour.

Due to the similarity between our results and the ones previously described [28,29], it is tempting to suggest that a similar phenomenon occurred in our cell lines. The presence of a few asynchronous peaks in viral RNA transcripts and in extracellular titres (Fig. 3) may support this hypothesis, as the primers used may amplify viral RNA from both DIPs and standard viruses (although a deletion of the region amplified by the set of primers used cannot be ruled out).

## Conclusion and perspectives

Our study indicates that WT EC^IPNV^ and *pkr^-/-^* EC-PKR-C4^IPNV^ cells present most of the canonical characteristics of persistently infected cells. A striking feature of these cell lines is the oscillatory pattern of extracellular titres and of intracellular viral RNA levels in both cell lines over the course of passages. Our results further suggest that the host’s type I IFN response is not triggered during persistent infection and that PKR does not play a major role in the regulation of this persistence phenomenon. Taken together, these results suggest that persistent IPNV has evolved strategies to evade the host innate immune response, while maintaining a relatively low replication level.

To go further, it would be interesting to confirm these findings by studying the impact of IPNV persistent infection on the cellular transcriptome over time using a whole transcriptome sequencing approach. An RNA-Seq study would also provide novel insights into alternative host’s pathways that are modulated during persistent infections, thereby helping identify additional mechanisms underlying IPNV persistence. Another future research axis will be to investigate the presence of DIPs in the supernatants to ultimately study whether our system follows a predator–prey dynamic. In particular, it would be interesting to develop an assay to quantify DIPs in our collected supernatants, either by using centrifugation in caesium chloride gradients [16,28], by measuring the CPE-suppressing activity of these supernatants against homologous and heterologous viruses [13,28] and/or by sequencing of the viral genome from the different particle types present in the supernatant. The presence of soluble anti-viral factors could also be assessed by testing the antiviral activity of the supernatants using size-exclusion techniques to remove virions.

## Supplementary material

10.1099/jgv.0.002283 Supplementary Material 1.

## Abbreviations

CHSE: Chinook salmon embryo
CPE: cytopathic effect
DIP: defective interfering particle
GMEM: Glasgow’s modified Eagle’s medium
IHNV: infectious hematopoietic necrosis virus
IPNV: infectious pancreatic necrosis virus
RT-qPCR: real-time quantitative PCR
