# Supplementary material for: Characterization of cell lines persistently infected with infectious pancreatic necrosis virus
Source: J Gen Virol. 2026 Jun 18;107(6):002283. doi: 10.1099/jgv.0.002283 (PMC13278376; doi:10.1099/jgv.0.002283)
Supplement: Supplementary Material 1. [file jgv-107-02283-s001.pdf]

Clones	Primers	Indel	Chromatogramme
EC-PKR-C4-GFP(+) = EC[IPNV]	PKR-gen-F	WT	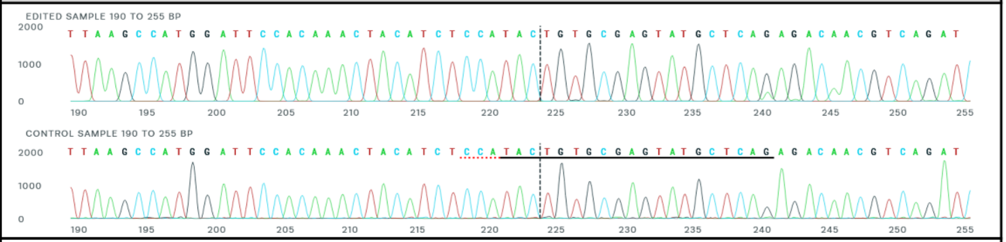
	PKR-gen-R	WT	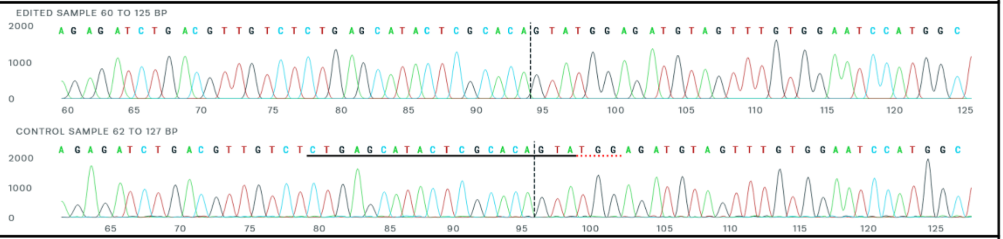
EC-PKR-C4-GFP(-) = EC-PKR-C4[IPNV]	PKR-gen-F	+1	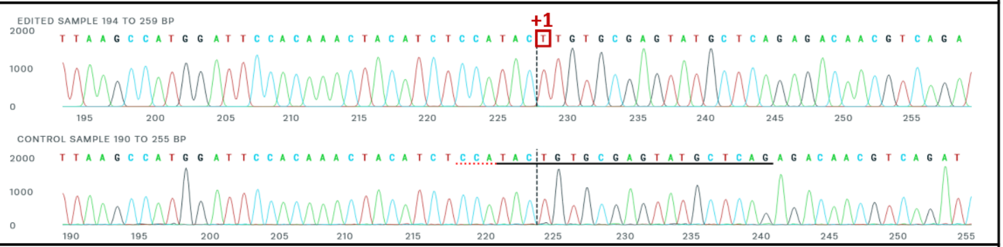
	PKR-gen-R	+1	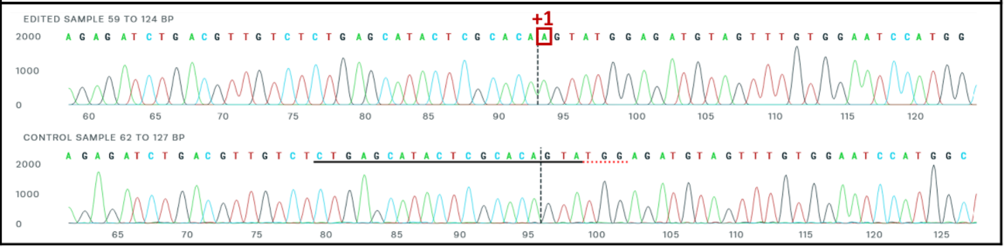

Figure S1: Alignment of chromatograms from EC^{IPNV} and EC-PKR-C4^{IPNV} with EC (WT) cell line. Chromatograms showing edited and wild-type (control) sequences in the region around the sequence targeted by sgRNA-PKR1 from EC^{IPNV} (EC-PKR-C4-GFP(+)) and EC-PKR-C4^{IPNV} (EC-PKR-C4-GFP(-)). The horizontal black line represents the guide sequence; the horizontal red dotted line corresponds to the PAM site; the vertical black dotted line represents the actual cut site. The red boxes show inserted nucleotides. Alignments were obtained using Synthego ICE Analysis tool [29].

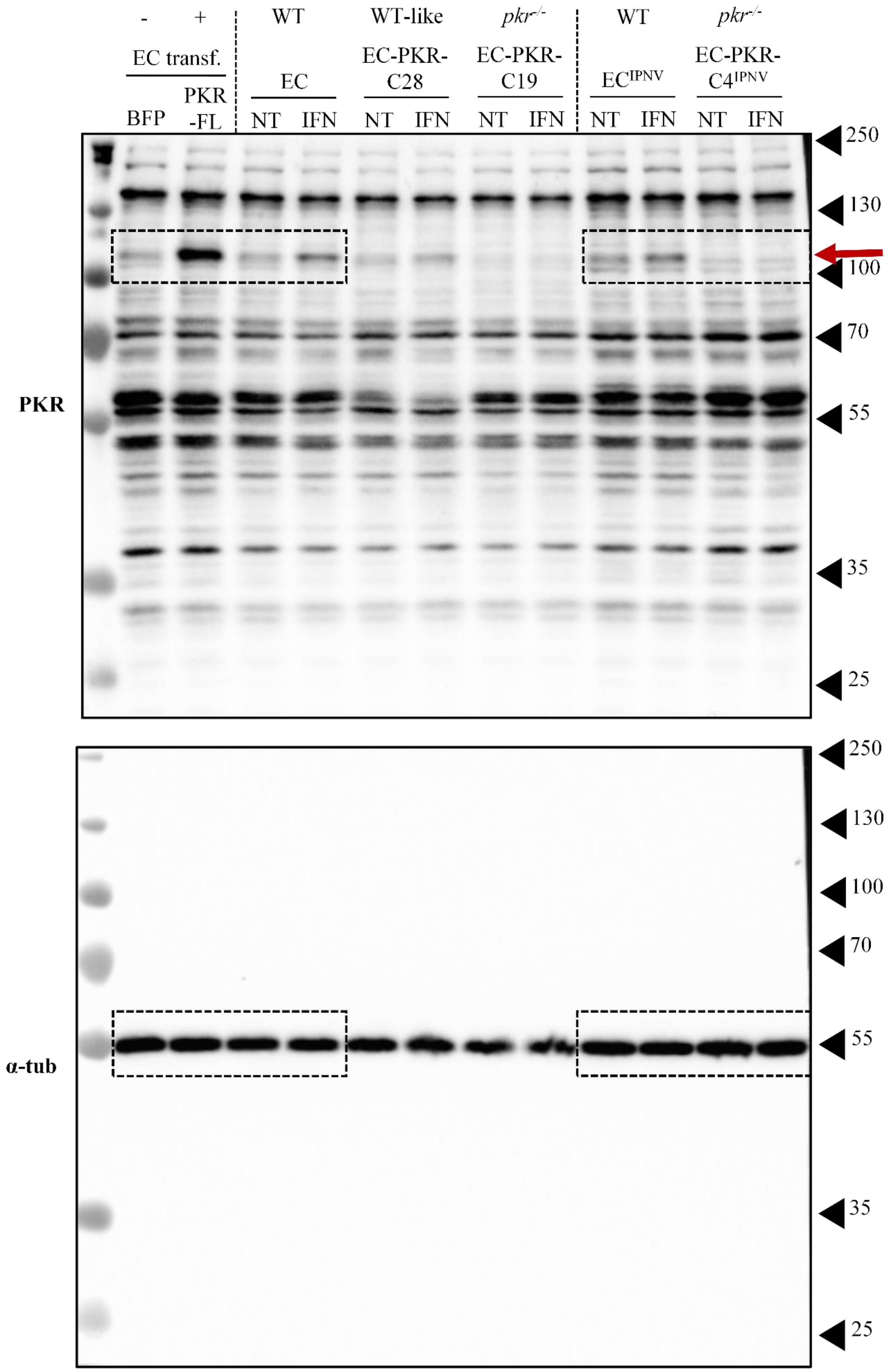

Figure S2: Original full-length blots used in Figure 2C. Regions corresponding to the cropped images are surrounded by a dotted line. The red arrow shows the band corresponding to PKR-FL.

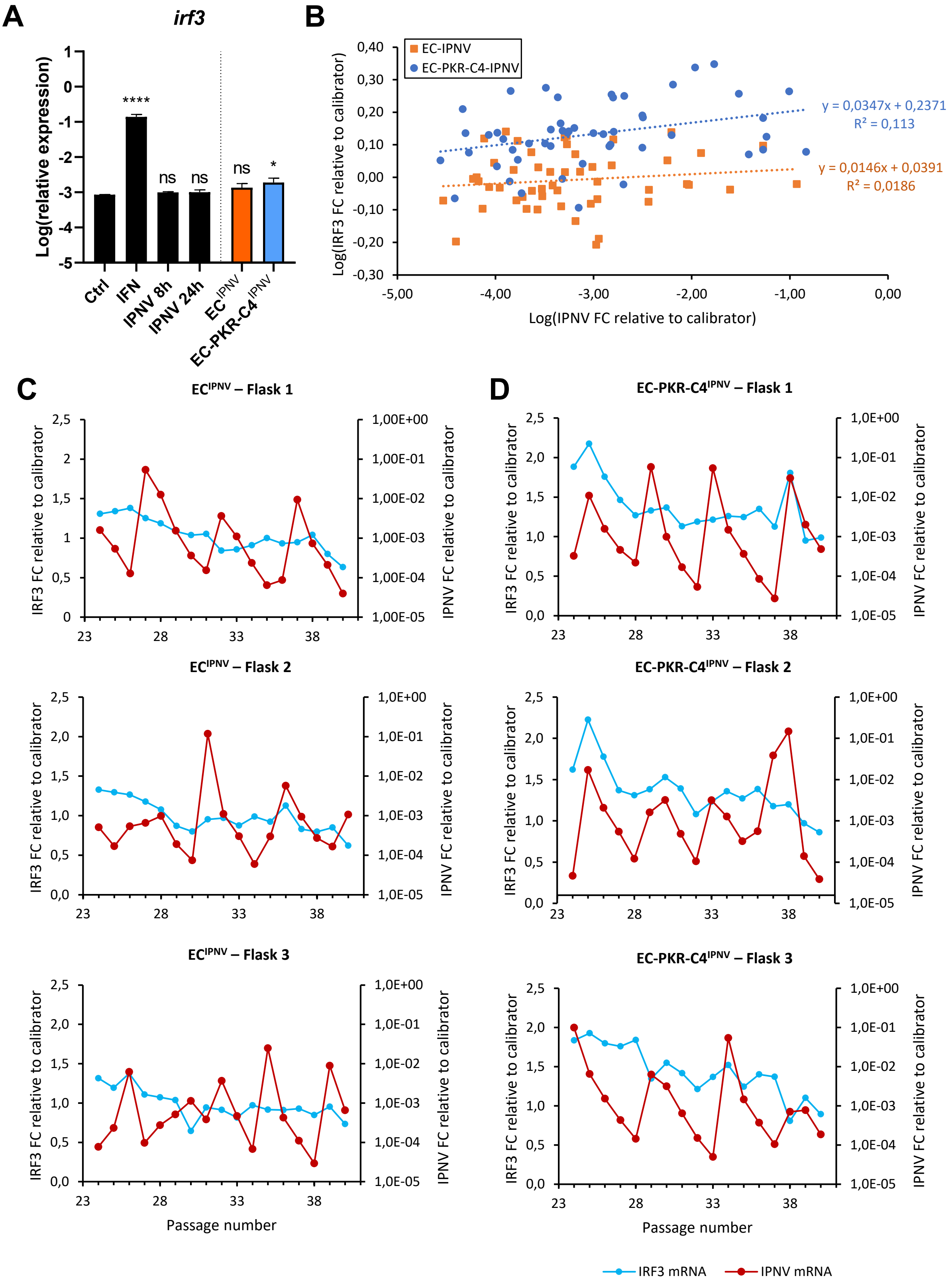

Figure S3: irf3 expression levels in persistently IPNV-infected cells (A) Dotplot showing log-transformed *ipnv* mRNA levels (represented as fold change relative to calibrator) as a function of log-transformed irf3 mRNA levels in persistently IPNV-infected EC^{IPNV} and $EC\text{-}PKR\text{-}C4^{IPNV}$ cells. Distinct linear regressions were performed on EC^{IPNV} and $EC\text{-}PKR\text{-}C4^{IPNV}$ datasets. (B,C) Graphs showing ipnv and *irf3* mRNA levels over the course of passages in each individual flasks of EC^{IPNV} (B) and $EC\text{-}PKR\text{-}C4^{IPNV}$ (C).

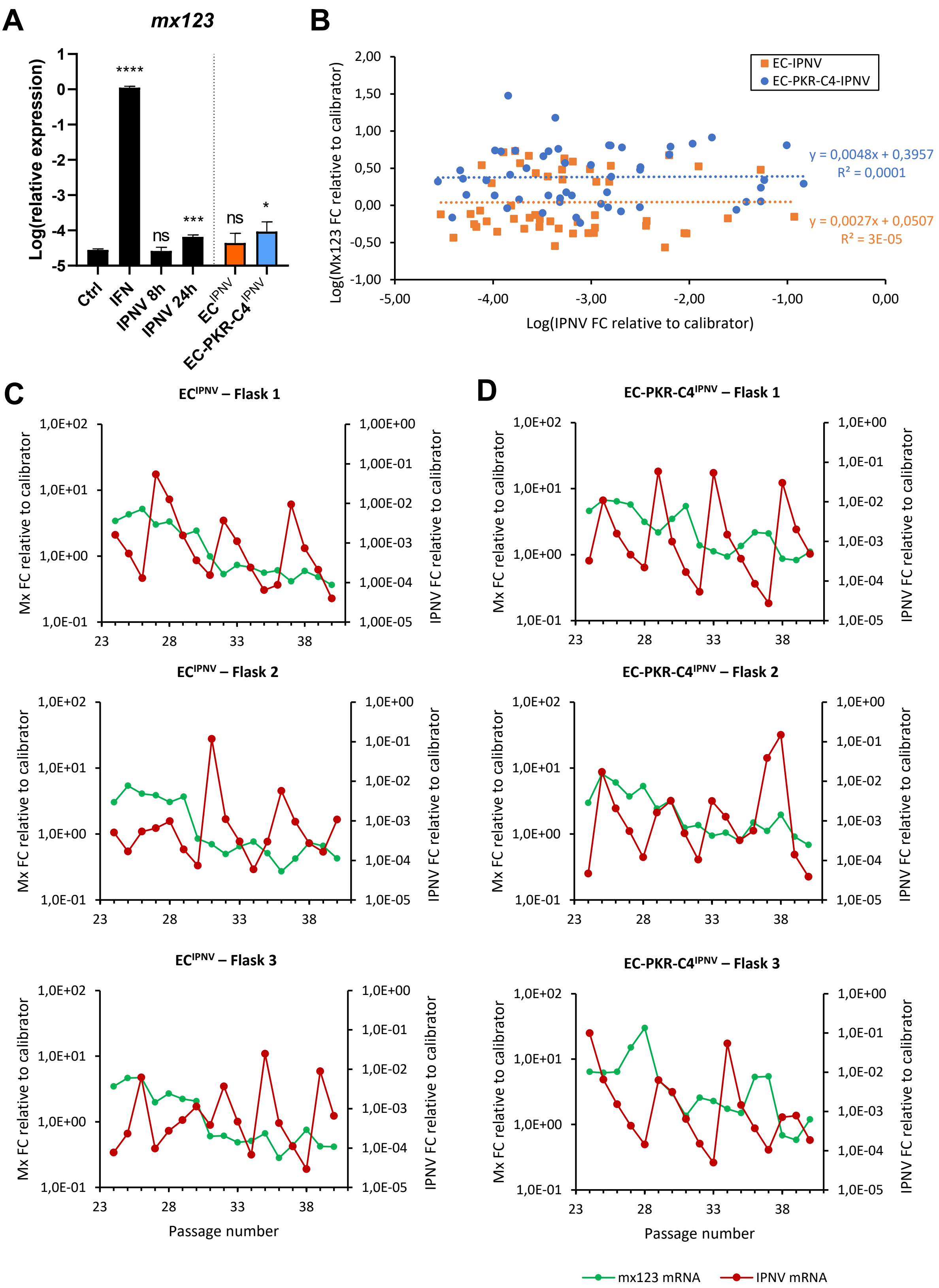

Figure S4: mx123 expression levels in persistently IPNV-infected cells. (A) Dotplot showing log-transformed *ipnv* mRNA levels (represented as fold change relative to calibrator) as a function of log-transformed mx123 mRNA levels in persistently IPNV-infected EC^{IPNV} and $EC\text{-}PKR\text{-}C4^{IPNV}$ cells. Distinct linear regressions were performed on EC^{IPNV} and $EC\text{-}PKR\text{-}C4^{IPNV}$ datasets. (B,C) Graphs showing ipnv and mx123 mRNA levels over the course of passages in each individual flasks of EC^{IPNV} (B) and $EC\text{-}PKR\text{-}C4^{IPNV}$ (C).

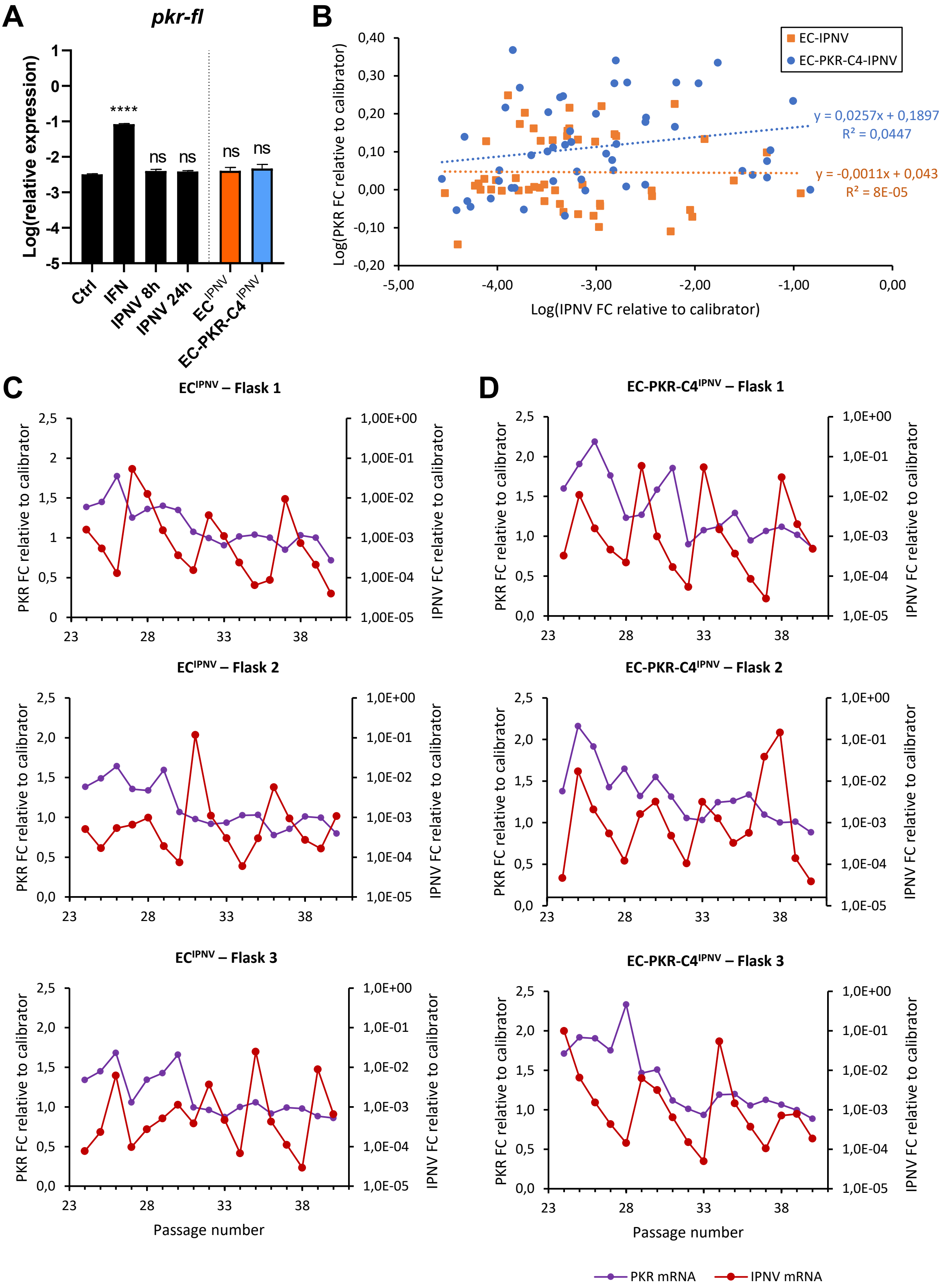

Figure S5: PKR expression levels in persistently IPNV-infected cells. (A) Dotplot showing log-transformed *ipnv* mRNA levels (represented as fold change relative to calibrator) as a function of log-transformed pkr-fl mRNA levels in persistently IPNV-infected EC^{IPNV} and EC-PKR-C4^{IPNV} cells. Distinct linear regressions were performed on EC^{IPNV} and EC-PKR-C4^{IPNV} datasets. (B,C) Graphs showing ipnv and pkr-fl mRNA levels over the course of passages in each individual flasks of EC^{IPNV} (B) and EC-PKR-C4^{IPNV} (C).

Reference

29. **Conant D, Hsiau T, Rossi N, Oki J, Maures T, *et al***. Inference of CRISPR edits from sanger trace data. *CRISPR J* 2022;5:123–130. 10.1089/crispr.2021.0113
